# Supplementary material for: Diffusion probabilistic model based accurate and high-degree-of-freedom metasurface inverse design
Source: Nanophotonics. 2023 Oct 4;12(20):3871–81. doi: 10.1515/nanoph-2023-0292 (PMC11501780; doi:10.1515/nanoph-2023-0292)
Supplement: Supplementary file 1 — Supplementary Material Details [file j_nanoph-2023-0292_suppl_001.pdf]

Supplementary material

Diffusion Probabilistic Model Based Accurate and High-Degree-of-Freedom Metasurface Inverse Design

Zezhou Zhang, Chuanchuan Yang, Yifeng Qin*, Hao Feng, Jiqiang Feng, Hongbin Li*

1. Detailed Formula derivations

Here, we provide the detailed derivations of Equation 3, Equation 6-7, Equation 8, and Equation 10 based on diffusion probabilistic theory. [1]

1.1. Equation 3

By using the reparameterization trick in Equation 1 and defining $\alpha_t = 1-\beta_t$, $\bar{\alpha}_t = \prod_{s=1}^{t}\alpha_s$, the noisy meta-atom matrix x_t at any time step t can be directly expressed as a closed-form formula based on x_0:

$$\begin{aligned}
x_t &= \sqrt{1-\beta_t}x_{t-1} + \sqrt{\beta_t}\cdot\epsilon \\
&= \sqrt{\alpha_t}x_{t-1} + \sqrt{1-\alpha_t}\cdot\epsilon \\
&= \sqrt{\alpha_t}(\sqrt{\alpha_{t-1}}x_{t-2} + \sqrt{1-\alpha_{t-1}}\cdot\epsilon) + \sqrt{1-\alpha_t}\cdot\epsilon \\
&= \sqrt{\alpha_t\alpha_{t-1}}x_{t-2} + \sqrt{1-\alpha_t\alpha_{t-1}}\cdot\epsilon \\
&\dots \\
&= \sqrt{\bar{\alpha}_t}x_0 + \sqrt{1-\bar{\alpha}_t}\cdot\epsilon
\end{aligned} \tag{S1}$$

1.2. Equation 6-7

$$\begin{aligned}
\mathbb{E}_{q(x_{1:T}\mid x_0)}\left[-\log p_\theta(x_0)\right] &\le \mathbb{E}_{q(x_{1:T}\mid x_0)}\left[-\log p_\theta(x_0)\right] + D_{KL}(q(x_{1:T}\mid x_0)\,\|\,p_\theta(x_{1:T}\mid x_0)) \\
&= \mathbb{E}_{q(x_{1:T}\mid x_0)}\left[-\log p_\theta(x_0) + \log\frac{q(x_{1:T}\mid x_0)}{p_\theta(x_{1:T}\mid x_0)}\right] \\
&= \mathbb{E}_{q(x_{1:T}\mid x_0)}\left[-\log p_\theta(x_0) + \log\frac{q(x_{1:T}\mid x_0)}{(p_\theta(x_0\mid x_{1:T})\,p_\theta(x_{1:T}))/p_\theta(x_0)}\right] \\
&= \mathbb{E}_{q(x_{1:T}\mid x_0)}\left[-\log p_\theta(x_0) + \log\frac{q(x_{1:T}\mid x_0)}{p_\theta(x_0, x_{1:T})/p_\theta(x_0)}\right] \\
&= \mathbb{E}_{q(x_{1:T}\mid x_0)}\left[-\log p_\theta(x_0) + \log\frac{q(x_{1:T}\mid x_0)}{p_\theta(x_{0:T})/p_\theta(x_0)}\right] \\
&= \mathbb{E}_{q(x_{1:T}\mid x_0)}\left[-\log p_\theta(x_0) + \log\frac{q(x_{1:T}\mid x_0)}{p_\theta(x_{0:T})} + \log p_\theta(x_0)\right] \\
&= \mathbb{E}_{q(x_{1:T}\mid x_0)}\left[-\log\frac{p_\theta(x_{0:T})}{q(x_{1:T}\mid x_0)}\right] =: \mathrm{L}
\end{aligned} \tag{S2}$$

The variational lower bound L can be further simplified as L_T, L_{t-1}, L_0 :

$$
\begin{aligned}
L &= \mathbb{E}_{q(x_{1:T} \mid x_0)}\left[-\log \frac{p_\theta(x_T)\prod_{t=1}^{T} p_\theta\left(x_{t-1} \mid x_t\right)}{\prod_{t=1}^{T} q\left(x_t \mid x_{t-1}\right)}\right] \\
&= \mathbb{E}_{q(x_{1:T} \mid x_0)}\left[-\log p_\theta\left(x_T\right) - \sum_{t\geq 1}\log \frac{p_\theta\left(x_{t-1} \mid x_t\right)}{q\left(x_t \mid x_{t-1}\right)}\right] \\
&= \mathbb{E}_{q(x_{1:T} \mid x_0)}\left[-\log p_\theta\left(x_T\right) - \sum_{t\geq 2}\log \frac{p_\theta\left(x_{t-1} \mid x_t\right)}{q\left(x_t \mid x_{t-1}\right)} - \log \frac{p_\theta\left(x_0 \mid x_1\right)}{q\left(x_1 \mid x_0\right)}\right] \\
&= \mathbb{E}_{q(x_{1:T} \mid x_0)}\left[-\log p_\theta\left(x_T\right) - \sum_{t\geq 2}\log \frac{p_\theta\left(x_{t-1} \mid x_t\right)}{q\left(x_t \mid x_{t-1}, x_0\right)} - \log \frac{p_\theta\left(x_0 \mid x_1\right)}{q\left(x_1 \mid x_0\right)}\right] \\
&= \mathbb{E}_{q(x_{1:T} \mid x_0)}\left[-\log p_\theta\left(x_T\right) - \sum_{t\geq 2}\log \frac{p_\theta\left(x_{t-1} \mid x_t\right)q(x_{t-1} \mid x_0)}{q\left(x_{t-1} \mid x_t, x_0\right)q(x_t \mid x_0)} - \log \frac{p_\theta\left(x_0 \mid x_1\right)}{q\left(x_1 \mid x_0\right)}\right] \\
&= \mathbb{E}_{q(x_{1:T} \mid x_0)}\left[-\log p_\theta\left(x_T\right) - \sum_{t\geq 2}\log \frac{p_\theta\left(x_{t-1} \mid x_t\right)}{q\left(x_{t-1} \mid x_t, x_0\right)} - \log \frac{q\left(x_1 \mid x_0\right)}{q\left(x_T \mid x_0\right)} - \log \frac{p_\theta\left(x_0 \mid x_1\right)}{q\left(x_1 \mid x_0\right)}\right] \\
&= \mathbb{E}_{q(x_{1:T} \mid x_0)}\left[-\log \frac{p_\theta\left(x_T\right)}{q\left(x_T \mid x_0\right)} - \sum_{t\geq 2}\log \frac{p_\theta\left(x_{t-1} \mid x_t\right)}{q\left(x_{t-1} \mid x_t, x_0\right)} - \log p_\theta\left(x_0 \mid x_1\right)\right] \\
&= \mathbb{E}_{q(x_T \mid x_0)}\left[-\log \frac{p_\theta\left(x_T\right)}{q\left(x_T \mid x_0\right)}\right] - \sum_{t\geq 2}\mathbb{E}_{q(x_t, x_{t-1} \mid x_0)}\left[\log \frac{p_\theta\left(x_{t-1} \mid x_t\right)}{q\left(x_{t-1} \mid x_t, x_0\right)}\right] - \mathbb{E}_{q(x_1 \mid x_0)}\left[\log p_\theta\left(x_0 \mid x_1\right)\right] \\
&= \underbrace{D_{KL}(q\left(x_T \mid x_0\right) \,\|\, p_\theta\left(x_T\right))}_{L_T} + \sum_{t\geq 2}\underbrace{\mathbb{E}_{q(x_t \mid x_0)}\left[D_{KL}(q\left(x_{t-1} \mid x_t, x_0\right) \,\|\, p_\theta\left(x_{t-1} \mid x_t\right))\right]}_{L_{t-1}} - \underbrace{\mathbb{E}_{q(x_1 \mid x_0)}\left[\log p_\theta\left(x_0 \mid x_1\right)\right]}_{L_0}
\end{aligned} \tag{S3}
$$

where L_T is a constant without any learnable parameters, and thus it can be ignored during the training process. L_0 represents the single step of reconstructing x_0 after obtaining x_1 and has a minor impact on L. And therefore, the primary determinant of L is L_{t-1}. It is worth noting that the inclusion of an additional condition x_0 in $q\left(x_t \mid x_{t-1}\right) = q(x_t \mid x_{t-1}, x_0)$ does not alter the results due to the Markov Property.

1.3. Equation 8

In L_{t-1}, the posterior $q\left(x_{t-1} \mid x_t, x_0\right)$ can be expressed as：

$$q\left(x_{t-1} \mid x_t, x_0\right) = \mathcal{N}\left(x_{t-1}; \tilde{\boldsymbol{\mu}}_t\left(x_t, x_0\right), \tilde{\beta}_t\mathbf{I}\right) \tag{S4}$$

Bayes’ rule can be used to extend the formula:

$$
\begin{aligned}
q(x_{t-1} \mid x_t, x_0) &= q(x_t \mid x_{t-1}, x_0)\frac{q(x_{t-1} \mid x_0)}{q(x_t \mid x_0)} \\
&= \frac{\mathcal{N}\left(x_t; \sqrt{\alpha_t}x_{t-1}, \beta_t \mathbf{I}\right)\mathcal{N}\left(x_{t-1}; \sqrt{\bar{\alpha}_{t-1}}x_0, (1-\bar{\alpha}_{t-1})\mathbf{I}\right)}{\mathcal{N}\left(x_t; \sqrt{\bar{\alpha}_t}x_0, (1-\bar{\alpha}_t)\mathbf{I}\right)} \\
&\propto \exp\Big(-\frac{1}{2}\Big(\frac{(x_t-\sqrt{\alpha_t}x_{t-1})^2}{\beta_t}+\frac{(x_{t-1}-\sqrt{\bar{\alpha}_{t-1}}x_0)^2}{1-\bar{\alpha}_{t-1}}-\frac{(x_t-\sqrt{\bar{\alpha}_t}x_0)^2}{1-\bar{\alpha}_t}\Big)\Big) \\
&= \exp\Big(-\frac{1}{2}\Big(\frac{x_t^2-2\sqrt{\alpha_t}x_t x_{t-1}+\alpha_t x_t^2 x_{t-1}^2}{\beta_t}+\frac{x_{t-1}^2-2\sqrt{\bar{\alpha}_{t-1}}x_0 x_{t-1}+\bar{\alpha}_{t-1}x_0^2}{1-\bar{\alpha}_{t-1}}-\frac{(x_t-\sqrt{\bar{\alpha}_t}x_0)^2}{1-\bar{\alpha}_t}\Big)\Big) \\
&= \exp\Big(-\frac{1}{2}\Big(\Big(\frac{\alpha_t}{\beta_t}+\frac{1}{1-\bar{\alpha}_{t-1}}\Big)x_{t-1}^2-\Big(\frac{2\sqrt{\alpha_t}}{\beta_t}x_t+\frac{2\sqrt{\bar{\alpha}_{t-1}}}{1-\bar{\alpha}_{t-1}}x_0\Big)x_{t-1}+C(x_t,x_0)\Big)\Big)
\end{aligned}
\tag{S5}
$$

where $C(x_t, x_0)$ can be ignored since it is irrelevant to x_{t-1}, and based on the formula of Gaussian function, variance and mean expressions can be derived as：

$$
\tilde{\beta}_t = 1/\Big(\frac{\alpha_t}{\beta_t}+\frac{1}{1-\bar{\alpha}_{t-1}}\Big) = 1/\Big(\frac{\alpha_t-\bar{\alpha}_t+\beta_t}{\beta_t(1-\bar{\alpha}_{t-1})}\Big) = \frac{1-\bar{\alpha}_{t-1}}{1-\bar{\alpha}_t}\cdot\beta_t
\tag{S6}
$$

$$
\begin{aligned}
\boldsymbol{\mu}_t(x_t, x_0) &= \Big(\frac{\sqrt{\alpha_t}}{\beta_t}x_t+\frac{\sqrt{\bar{\alpha}_{t-1}}}{1-\bar{\alpha}_{t-1}}x_0\Big)/\Big(\frac{\alpha_t}{\beta_t}+\frac{1}{1-\bar{\alpha}_{t-1}}\Big) \\
&= \frac{\sqrt{\alpha_t}(1-\bar{\alpha}_{t-1})}{1-\bar{\alpha}_t}x_t+\frac{\sqrt{\bar{\alpha}_{t-1}}\beta_t}{1-\bar{\alpha}_t}x_0 \\
&= \frac{\sqrt{\alpha_t}(1-\bar{\alpha}_{t-1})}{1-\bar{\alpha}_t}x_t+\frac{\sqrt{\bar{\alpha}_{t-1}}\beta_t}{1-\bar{\alpha}_t}\Big(\frac{1}{\sqrt{\bar{\alpha}_t}}(x_t-\sqrt{1-\bar{\alpha}_t}\epsilon_t)\Big) \\
&= \frac{1}{\sqrt{\alpha_t}}\Big(x_t-\frac{1-\alpha_t}{\sqrt{1-\bar{\alpha}_t}}\epsilon_t\Big)
\end{aligned}
\tag{S7}
$$

We can define $\Sigma_\theta(x_t, t) = \tilde{\beta}_t$, and based on formula of KL divergence [2, 3]:

$$
\begin{aligned}
L_\theta &\propto \mathbb{E}_{q(x_t \mid x_0)}\left[D_{KL}(q(x_{t-1} \mid x_t, x_0) \,\|\, p_\theta(x_{t-1} \mid x_t))\right] \\
&= \mathbb{E}_{q(x_t \mid x_0)}\left[\frac{1}{2}\left[\log\frac{\det\tilde{\beta}_t}{\det\tilde{\beta}_t}-n+tr(\tilde{\beta}_t^{-1}\tilde{\beta}_t)+(\boldsymbol{\mu}_\theta(x_t,t)-\tilde{\boldsymbol{\mu}}_t(x_t,x_0))^T\tilde{\beta}_t^{-1}(\boldsymbol{\mu}_\theta(x_t,t)-\tilde{\boldsymbol{\mu}}_t(x_t,x_0))\right]\right] \\
&= \mathbb{E}_{q(x_t \mid x_0)}\left[\frac{1}{2}\left[\log 1-n+n+(\boldsymbol{\mu}_\theta(x_t,t)-\tilde{\boldsymbol{\mu}}_t(x_t,x_0))^T\tilde{\beta}_t^{-1}(\boldsymbol{\mu}_\theta(x_t,t)-\tilde{\boldsymbol{\mu}}_t(x_t,x_0))\right]\right] \\
&= \mathbb{E}_{q(x_t \mid x_0)}\left[\frac{1}{2\tilde{\beta}_t}\|\boldsymbol{\mu}_\theta(x_t,t)-\tilde{\boldsymbol{\mu}}_t(x_t,x_0)\|^2\right]
\end{aligned}
\tag{S8}
$$

where $\boldsymbol{\mu}_\theta$ is used to approximate $\tilde{\boldsymbol{\mu}}_t$, and can be expressed as：

$$
\boldsymbol{\mu}_\theta(x_t,t) = \frac{1}{\sqrt{\alpha_t}}\Big(x_t-\frac{1-\alpha_t}{\sqrt{1-\bar{\alpha}_t}}\epsilon_\theta(x_t,t)\Big)
\tag{S9}
$$

By plugging $\tilde{\boldsymbol{\mu}}_t$ and $\boldsymbol{\mu}_\theta$, the training process can be simplified as:

$$
\begin{aligned}
\arg\min_{\theta} L &= \arg\min_{\theta} \mathbb{E}_{q(x_t \mid x_0)}\left[\frac{1}{2\tilde{\beta}_t} \| \boldsymbol{\mu}_\theta\left(x_t, t\right) - \tilde{\boldsymbol{\mu}}_t(x_t, x_0) \|^2\right] \\
&= \arg\min_{\theta} \mathbb{E}_{q(x_t \mid x_0)}\left[\frac{1}{2\tilde{\beta}_t} \| \frac{1}{\sqrt{\alpha_t}}(x_t - \frac{1-\alpha_t}{\sqrt{1-\bar{\alpha}_t}}\epsilon_\theta(x_t, t)) - \frac{1}{\sqrt{\alpha_t}}(x_t - \frac{1-\alpha_t}{\sqrt{1-\bar{\alpha}_t}}\epsilon_t) \|^2\right] \\
&= \arg\min_{\theta} \mathbb{E}_{q(x_t \mid x_0)}\left[\frac{(1-\alpha_t)^2}{2\tilde{\beta}_t \alpha_t (1-\bar{\alpha}_t)} \| \epsilon_\theta(x_t, t) - \epsilon_t \|^2\right] \\
&\propto \arg\min_{\theta} \mathbb{E}_{q(x_t \mid x_0)}\left[\| \epsilon_\theta(x_t, t) - \epsilon_t \|^2\right]
\end{aligned} \tag{S10}
$$

1.4. Equation 10

x_{t-1} can be reparameterized according to Equation S4, Equation S6, Equation S7 and Equation 9:

$$
\begin{aligned}
x_{t-1} &= \tilde{\boldsymbol{\mu}}_t\left(x_t, x_0\right) + \tilde{\beta}_t \cdot z, \\
&= \frac{1}{\sqrt{\alpha_t}}\left(x_t - \frac{1-\alpha_t}{\sqrt{1-\bar{\alpha}_t}}\tilde{\epsilon}_\theta\left(x_t, t, c\right)\right) + \frac{1-\bar{\alpha}_{t-1}}{1-\bar{\alpha}_t} \cdot \beta_t \cdot z, z \sim \mathcal{N}(0,1)
\end{aligned} \tag{S11}
$$

2. MetaDiffusion Training and Generating Algorithm

Algorithm S1 MetaDiffusion Training

Input: pixelated geometry, corresponding S-Parameters and extra parameters Dataset $\{X, S, W1, H2, N2\}$, probability of unconditional training p_{uncond}, total step T

1: **repeat**
2: Sample $x_0 \sim X$
3: Sample condition $c \sim \{S, W1, H2, N2\}$
4: Randomly dropout condition $c \leftarrow \varnothing$ with probability p_{uncond}
5: Sample $t \sim \text{Uniform}(\{1, .., T\})$, and $\epsilon \sim \mathcal{N}(0, I)$
6: Compute x_t using closed form Equation 3: $x_t = \sqrt{\bar{\alpha}_t}x_0 + \sqrt{1-\bar{\alpha}_t}\epsilon$
7: Take gradient descent step on $\nabla_\theta \left\|\epsilon - \epsilon_\theta\left(x_t, c, t\right)\right\|^2$
8: **until** converged

During the training phase, as shown in Algorithm 1, the meta-atom x_0 is first sampled from the training set. Then, its corresponding S-Parameter and extra parameters (meta-atom size $W1$, thickness $H2$, material refractive index $N2$) are sampled from the training set and concatenated to a 1×55 vector as a condition input. Then, with a probability of 10%, we replace the condition input with the unconditional marker 0. Since the actual training is performed on the entire batch, this is equivalent to randomly selecting 10% of the samples for unconditional training. For each sample, we randomly select a time step to perform forward diffusion and add noise according to Equation 3 to directly obtain the noisy data x_t at the corresponding time step. We save the added noise ϵ as the training label for the neural network.

The noisy data x_t, condition input, and time step are jointly input into the neural network. The neural network outputs the estimated noise result ϵ_θ. The mean squared error (MSE) between the neural network output ϵ_θ and the noise label ϵ is calculated as the model loss and used to train the MetaDiffusion network through backpropagation.

Algorithm S2 MetaDiffusion Generating

Input: required S-Parameters and extra parameters condition c, guidance strength w, well-trained network ϵ_θ

Sample $x_T \sim \mathcal{N}(0, I)$

for $t = T, ..., 1$ **do**

  Mix conditional and unconditional results $\tilde{\epsilon}_t = (1 + w)\epsilon_\theta(x_t, c) - w\epsilon_\theta(x_t, 0)$

  Sample $z \sim \mathcal{N}(0, I)$ if $t > 1$, else $z = 0$

  Compute x_{t-1} as Equation 10: $x_{t-1} = \frac{1}{\sqrt{\alpha_t}}\left(x_t - \frac{1-\alpha_t}{\sqrt{1-\bar{\alpha}_t}}\tilde{\epsilon}_t\right) + \tilde{\beta}_t z$

end for

return x_0 as generated geometry

During the generation phase, as shown in Algorithm 2, we first sample x_T from a Gaussian distribution. Then, we input x_T and the condition c into the network to obtain the conditional prediction result. We also input x_T and the unconditional marker 0 into the network to obtain the unconditional prediction result. The conditional $\epsilon_\theta(x_T, s)$ and unconditional $\epsilon_\theta(x_T)$ predictions are mixed in a ratio of w according to Equation 9 as the final prediction of the network. And the final prediction is used in Equation 10 to obtain x_{T-1}, and the above process is repeated from time step $t = T$ to time step $t = 1$. As the time step decreases, we gradually obtain generated results with lower noise and the distribution gradually approaches the target distribution. When $t = 1$, we set $z = 0$ and obtain the output new meta-atom x_0.

3. MetaDiffusion Network Structure

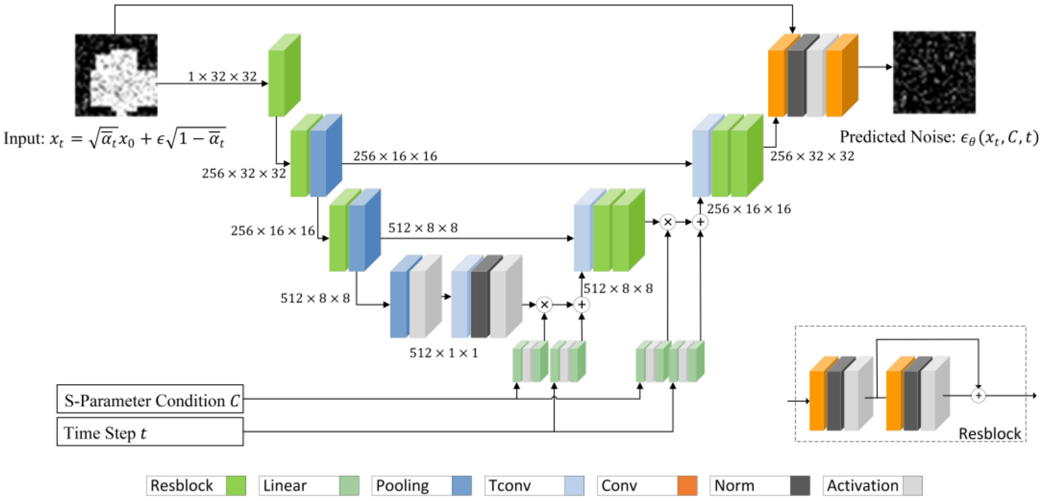

Figure S1. Specific Network Structure

Starting from the input, we use residual blocks to extract features from the original hypersurface structure x_t with input dimensions of (1×32×32) to obtain dimensions of (256×32×32). Then, we use two downsampling blocks to reduce the tensor dimensions to (512×8×8) and finally use a pooling layer with a GELU to vectorize the data (512×1×1) as the bottleneck of the U-Net structure. It is worth noting that during the downsampling process, as the matrix resolution decreases, the number of channels increases with the aim of extracting multi-dimensional features during compression. Then, we use three upsampling blocks to gradually increase the dimensions to (256×32×32). And in detail, for the first upsampling blocks, Transposed convolution with kernel size 8 and stride size 8 is used, followed by Group Norm and ReLU activation. For followed two upsampling blocks, Transposed convolutions with kernel size 2 and stride size 2 is used. Each upsampling block receives residual connections from the corresponding downsampling block. At the same time, the time step t and condition c are processed by the feature extraction layer to obtain embedded features. The embedded features of the condition are then input into the upsampling module to guide the model in estimating according to the current time step t and corresponding condition. Finally, the output of the upsampling block is fused with skip connections from the original input x_0 to further reduce information loss during downsampling and upsampling processes. After two convolutional layers (kernel size 3, stride 1, padding 1), the number of channels is reduced to a single channel as the final output, and Group Norm and ReLU activation are used in this final layer.

Each downsampling module first uses a residual block for feature extraction, followed by a pooling layer responsible for reducing dimensions. The upsampling module uses a transposed convolutional layer to increase dimensions, followed by two residual blocks for feature recovery. The embedded feature extraction layer used for preprocessing time step t and condition c consists of two fully connected layers with a GELU activation function in between. The residual block consists of two convolutional layers (kernel size 3, stride 1, padding 1), Batch Normalization, and GELU activation, where the output of the first convolutional block is added to the output of the second convolutional block as skip connections and divided by 1.414 to get output.

4. Hyperparameters

β_t represents the offset of the distribution at time step t and can be intuitively understood as the amount of noise added at the t-th time step. According to Equation 3 $x_t = \sqrt{\bar{\alpha}_t}\, x_0 + \epsilon\sqrt{1-\bar{\alpha}_t}$, where $\bar{\alpha}_t = \prod_{s=1}^{t}\left(1-\beta_s\right)$, it is necessary to continuously increase β_t in order to make $\sqrt{\bar{\alpha}_T}$ the mean of x_T approaching 0, and the variance $\sqrt{1-\bar{\alpha}_T}$ approaching 1. In the early stage of the noise addition process, the meta-atom pattern matrix contains less noise, so a small amount of noise will have a significant impact, leading to a rapid decline in the included information. In the later stages, as more noise has already been introduced, the impact of newly added noise

decreases. Therefore, under the condition of a consistent need for adding noise, less noise is added in the initial rounds and more noise is added later to slow down the rate of information loss. Thus, β_1 starts from 1e-4 and linearly increases to $\beta_T = 0.02$. Additionally, the selection of the total time step T involves a trade-off, with a larger value resulting in higher generation quality but requiring longer inference time. In this study, for the sake of generality, we simply chose T = 500 without further fine-tuning. However, in future work, if we conduct experimentation to search for the optimal hyperparameter T, it is possible to achieve even better results.

For generality, we simply use commonly used settings for the training parameters of the diffusion model without further optimization through careful selection, to demonstrate that our method has good robustness and can easily be generalized to different data scenarios. Specifically, we use Adam as an optimizer with a learning rate of 1e-4, simple linear decay and a batch size of 64. Following widely used standards, we divided the dataset into training, validation, and test sets in an 8:1:1 ratio. The training set is used for model training, the validation set is used to evaluate the training effect of each round during the training process, and the test set is used for the final model performance comparison. We use the 36×36 two-dimensional Boolean matrix in the upper left corner of the pixel matrix in the dataset as the original structure information x. We sample 26 points from both the real and imaginary parts of the corresponding transmission response and concatenate them into a 1×52 one-dimensional vector s. The S-Parameter vector then is concatenated with a 1×3 one-dimensional vector I representing structure parameters (thickness, refractive index, substrate size) to form a 1×55 one-dimensional vector as input condition c. The goal of the generation network is to generate a meta-atom structure x' that meets the requirements of the condition c.

5. Surrogate Solver PNN

PNN [4] is composed of multiple convolutional layers and an NTN layer. [5] The NTN layer is responsible for extracting information from the one-dimensional parameter matrix and uses multiple slices to capture more intrinsic information from the one-dimensional vector compared to a fully connected layer. The convolutional layers are responsible for extracting features from the two-dimensional free structure matrix. After fusing the one-dimensional and two-dimensional features, multiple convolutional layers are used for further processing. Finally, a fully connected layer regresses the real or imaginary part of the frequency response.

We use the two-dimensional free-form structure matrix and the one-dimensional extra parameter vector as inputs, where the one-dimensional extra parameter vector is used to specify the material, size, and thickness of meta-atoms. The real and imaginary parts of S-Parameters are used as

labels to train two networks to predict the real and imaginary parts separately. The PNN network is trained on the training set, and the validation set is used as a monitoring during the training process to evaluate the convergence and overfitting. Finally, the trained PNN is applied to the test set for performance evaluation. We use mean squared error (MSE) as the loss function:

$$L_{PNN} = \frac{1}{N}\sum_{n=1}^{N}\left(s_n - s_n^{'}\right)^2,$$

where s_n represents the ground-truth label obtained by CST simulation and $s_n^{'}$ represents the result predicted by PNN.

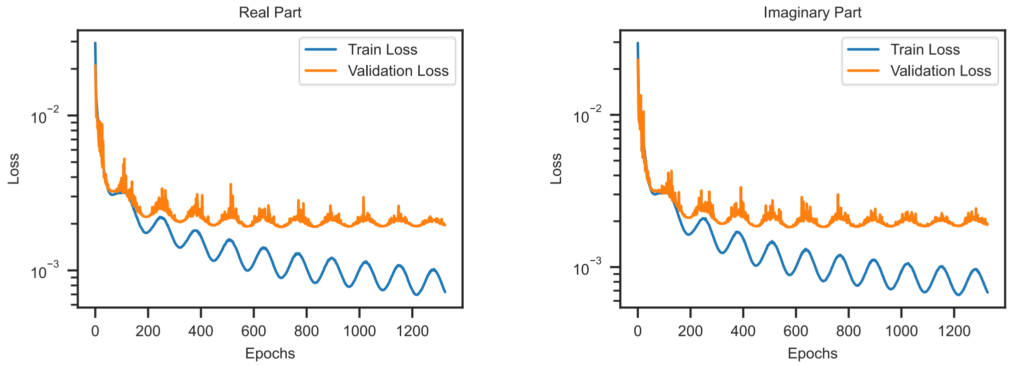

Figure S2. Loss values on the training and validation sets during the training process of the forward prediction network PNN.

Figure S1 shows the error on the training and validation sets during the training process. We use a cosine learning rate adjustment strategy during training to help escape local optima, so the loss value fluctuates periodically with the learning rate. However, it can still be clearly seen that the network gradually converges on the validation set, indicating that the network has been sufficiently trained. We apply the trained network to the test set for performance evaluation and obtain an error of 0.001835 for the real part and 0.001789 for the imaginary part. In addition, in Figure S2, we randomly select samples and use PNN for forward prediction and compare it with the simulated true values. The prediction results of the PNN network are in good agreement with the true results obtained by numerical simulations.

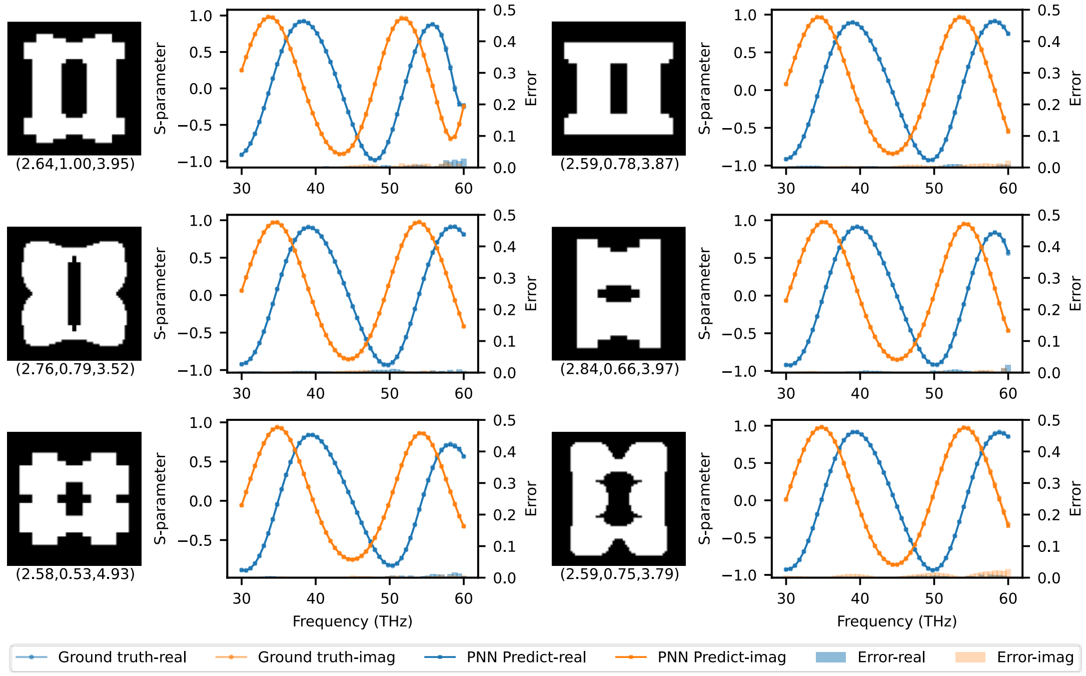

Figure S3. Qualitative examples of PNN.

Table S1 presents the MSE errors of MetaDiffusion, GAN-based and VAE-based models, comparing them with the MSE error of PNN. From the results, it can be observed that the potential errors introduced by PNN are significantly smaller than the performance differences among MetaDiffusion, GAN-based and VAE-based models. Therefore, PNN is accurate enough to evaluate the performance difference among MetaDiffusion, GAN-based and VAE-based models.

Table S1: The Test error of Models

**Model**	**MSE**
PNN (Real part)	0.001835
PNN (Imaginary part)	0.001789
PNN (Mean of two parts)	0.001812
SLMGAN	0.01084
WGAN-GP	0.01466
cVAE	0.01675
MetaDiffusion (Proposed)	0.005743

Table S2 provides the training and inference time for PNN, as well as a comparison of the time required for simulation using the CST Studio Suite frequency domain solver on the same model

CPU. From the table, it can be observed that PNN requires 65 hours of training time. However, during inference, it shows significantly faster speeds compared to the CST solver. Therefore, when inference on a large number of meta-atoms, PNN is a more efficient method compared to the CST solver.

Table S2: Time Comparison of PNN and CST solver

Model	Training Time (1300 epochs)	Inference Time (per atom)
PNN	65 hours	0.001 seconds
CST Frequency domain Solver	-	149 seconds

6. Comparative Method

For SLMGAN with a simulator, since there is no official open-source implementation code, we reproduce it as accurately as possible based on the details provided in the article. To ensure the accuracy of the key step Sinkhorn divergence, we use the standard PyTorch API from the GeomLoss Library [6] for Sinkhorn divergence calculation. In addition, for the introduction of symmetric information, we implement it by adding the generated 2D structure to its transpose and then interpolating it as mentioned in the original paper. In terms of model architecture, the dataset in the original paper uses 18×18 matrix as the metasurface pattern representation. To adapt to our higher precision 64×64 pattern representation, we increase the upscale factor of the PixelShuffle module and the corresponding channel number of the Upsampling modules in the generator based on the original architecture. For the discriminator, we only adjust the parameters of the last convolutional kernel to adapt to the size difference. In addition, SLMGAN requires a pre-trained accurate forward network simulator to be introduced as one of the training errors. The well-trained PNN can be used directly since it is proven to be a reliable surrogate solver. Furthermore, we adopt the recommended training parameter settings from the article including a learning rate of 1e-2 and using Adam as an optimizer.

For WGAN-GP, a representative and open-source implementation [7] is used as the basic model structure. We adjust the convolutional kernels and fully connected layers to fit the size of the inputs of the dataset. In addition, we add extra feature extraction layers for the input condition vectors in both the generator and discriminator to further enhance the network’s capabilities.

For conditional VAE (cVAE), a representative work[8] is used as the basic model structure. We adjust the convolutional kernels and fully connected layers to fit the size of the inputs of the dataset.

7. Time Information of Models

Table S3 provides the training time information for each epoch of the four models. Additionally, the training time required for the four models to reach MAE errors of 0.05 and 0.03 on the validation set is also compared in the table. From Table S3, it can be observed that although MetaDiffusion takes longer time per epoch compared to the GAN-based methods, it requires fewer epochs to converge. Therefore, the total training time required for MetaDiffusion is shorter than the other two GAN-based methods when converging to the same error. On the other hand, while cVAE boasts a quicker per-epoch training speed, it settles at a higher error rate compared to both MetaDiffusion and the GAN-based methods, indicating its relatively weaker learning capacity. In conclusion, our proposed model, MetaDiffusion, proves to be efficient.

Table S3: Training Time of Models. The training datasize for an epoch is 174483*0.8≅139906. The missing values indicate that the validation error of the corresponding method could not consistently decrease below the corresponding error value.

Model	Time (per epoch)	Time (Val Loss descend to 0.05)	Time (Val Loss descend to 0.03)
MetaDiffusion	196 seconds	49 epochs * 196 = 9604 seconds	210 epochs * 196 = 41160 seconds
SLMGAN	159 seconds	362 epochs *159 = 57558 seconds	-
WGAN-GP	112 seconds	327 epochs * 112 = 36624 seconds	-
cVAE	44 seconds	-	-

In this study, our primary goal has been on the generation accuracy and training stability of the model, with optimization of the generation process not being our main pursuit. Specifically, we employ a single-timestep, sequential denoising method commencing from Gaussian noise to derive the generated structures. This necessitates T denoising steps, resulting in a generation speed of 0.43 seconds per atom, which is comparatively slower than that of SLMGAN, WGAN-GP, and cVAE, which have speeds of 4.7e-5, 7.8e-5, and 7.4e-5 seconds, respectively. Nevertheless, there exists potential to enhance the generation speed of MetaDiffusion. In forthcoming research, we envision integrating advanced techniques, such as a multi-step denoising strategy[9] during the generation phase, building upon the trained foundation of the MetaDiffusion model. This integration could substantially diminish the requisite denoising steps during generation, thereby markedly accelerating the generation process.

8. Transfer learning to frequency range outside of training data

Transfer learning approach can be used with a very small dataset to make the model generalize to frequency bands outside of the training data with minimal cost. For example, let's consider the 20-30 THz range. Firstly, we collect a small amount of labeled data for the 20-30 THz range (e.g., 1000 samples, which is approximately 0.07% of the original training data). Then, we fine-tune the pre-trained model with a learning rate (1e-5) one order of magnitude smaller than the

initial training rate. The model is fine-tuned for just 50 epochs (10 minutes of training time). On the test set, the MAE is 0.04095, and the MSE is 0.0033. Additionally, one-to-many generation for the 20-30 THz range are also demonstrated in Figure S1. The PNN used for the surrogate solver is also trained on over 50,000 labeled data points in the 20-30 THz range to ensure accuracy, with the MSE of 1.64e-6 on the test set.

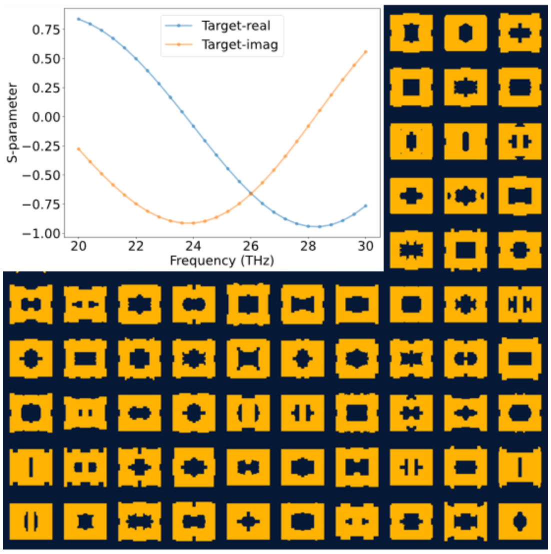

Figure S4. One to many Generations for the 20-30THz. Multiple unit cell structures that meet the given S-parameters target of 20-30THz are generated.

Reference

[1] J. Ho, A. Jain, P. Abbeel, in Advances in Neural Information Processing Systems, Curran Associates, Inc., 2020.

[2] J. Duchi, *Berkeley, California* 2007, *3*, 2325.

[3] C. Luo, arXiv preprint arXiv:2208.11970 2022.

[4] S. An, B. Zheng, M. Y. Shalaginov, H. Tang, H. Li, L. Zhou, J. Ding, A. M. Agarwal, C. Rivero-Baleine, M. Kang, K. A. Richardson, T. Gu, J. Hu, C. Fowler, H. Zhang, *Opt. Express* 2020, *28*, 31932.

[5] R. Socher, D. Chen, C. D. Manning, A. Ng, in *Advances in Neural Information Processing Systems*, Curran Associates, Inc., 2013.

[6] J. Feydy, T. Séjourné, F.-X. Vialard, S. Amari, A. Trouve, G. Peyré, in *Proceedings of the Twenty-Second International Conference on Artificial Intelligence and Statistics*, PMLR, 2019, pp. 2681–2690.

[7] J. Jiang, D. Sell, S. Hoyer, J. Hickey, J. Yang, J. A. Fan, *ACS Nano* 2019, *13*, 8872.

[8] W. Ma, F. Cheng, Y. Xu, Q. Wen, Y. Liu, Advanced Materials 2019, 31, 1901111.

[9] J. Song, C. Meng, S. Ermon, arXiv preprint arXiv:2010.02502 2020.
